# Study protocol: longitudinal observational study on frailty and mental health

**DOI:** 10.3389/fpubh.2026.1861495

**Published:** 2026-07-06

**Authors:** Petra Mikolič Brence, Branko Bregar, Katarina Vatovec, Tjaša Bertole, Suzana Oreški, Marjeta Ferlan Istinič, Lina Berlot, Matej Vinko

**Affiliations:** 1 Center for Mental Health, National Institute of Public Health, Ljubljana, Slovenia; 2 Faculty of Health Care, Angela Boškin, Jesenice, Slovenia; 3 Alma Mater Europaea University, Maribor, Slovenia; 4 Faculty of Medicine, University of Ljubljana, Ljubljana, Slovenia

**Keywords:** frailty, longitudinal study, mental health, older adults, public health

## Abstract

**Introduction:**

Frailty is a dynamic condition associated with increased vulnerability to adverse health outcomes in older adults. While previous research has primarily focused on deficit-based mental health factors, such as depression and loneliness, less is known about the role of positive mental health determinants, including wellbeing, resilience and social connectedness, in the development and progression of frailty. Understanding both risk and protective factors is essential for informing public health strategies aimed at promoting healthy aging. This study aims to examine the longitudinal relationship between mental health and frailty in a nationally sampled population of adults aged 50 years and older in Slovenia.

**Methods and analysis:**

This longitudinal observational cohort study will collect data at four time points over a 2-year period (January 2026-March 2028). A stratified random sample of community-dwelling adults aged 50–84 years will be drawn from the national population registry, with 5,000 individuals invited to participate in the first wave. Frailty, mental health and a set of social, psychological, and health-related factors will be assessed. Data will be analyzed using a combination of descriptive, inferential and longitudinal statistical methods to examine associations between frailty and mental health over time. Potential explanatory factors will also be explored within the longitudinal framework, and additional analyses will assess the impact of attrition.

## 1 Introduction

Frailty is a dynamic state or process characterized by functional decline and reduced physiological reserves of an individual, which can lead to adverse health outcomes (1). While the biopsychosocial model suggests that psychological and social factors contribute to this state, current research often fails to distinguish these as distinct longitudinal predictors of functional decline (2). By framing frailty as a discrete clinical outcome, we can explicitly evaluate how mental health trajectories—both deficit- and asset-based—drive or mitigate the progression of physiological vulnerability in older adults.

While traditional psychiatric epidemiology focuses on deficit-based models—primarily identifying depression and loneliness as drivers of functional decline—this approach overlooks the potential protective role of psychological assets (3, 4). Current evidence robustly links mental disorders to frailty risk, yet the longitudinal impact of positive mental health determinants, such as resilience and social inclusion, remains largely unquantified. Investigating these asset-based factors is essential for developing equitable public health interventions that move beyond symptom management toward health promotion in aging populations.

This study aims to elucidate the complex interplay between mental health and frailty to inform evidence-based public health interventions centered on social equity. Specifically, we will characterize the associations between these constructs in Slovenians aged 50 and older, determine the longitudinal directionality of these links, and investigate potential psychological, social, and health mechanisms underlying these associations.

## 2 Methods and analysis

### 2.1 Study design

**Figure 1:**
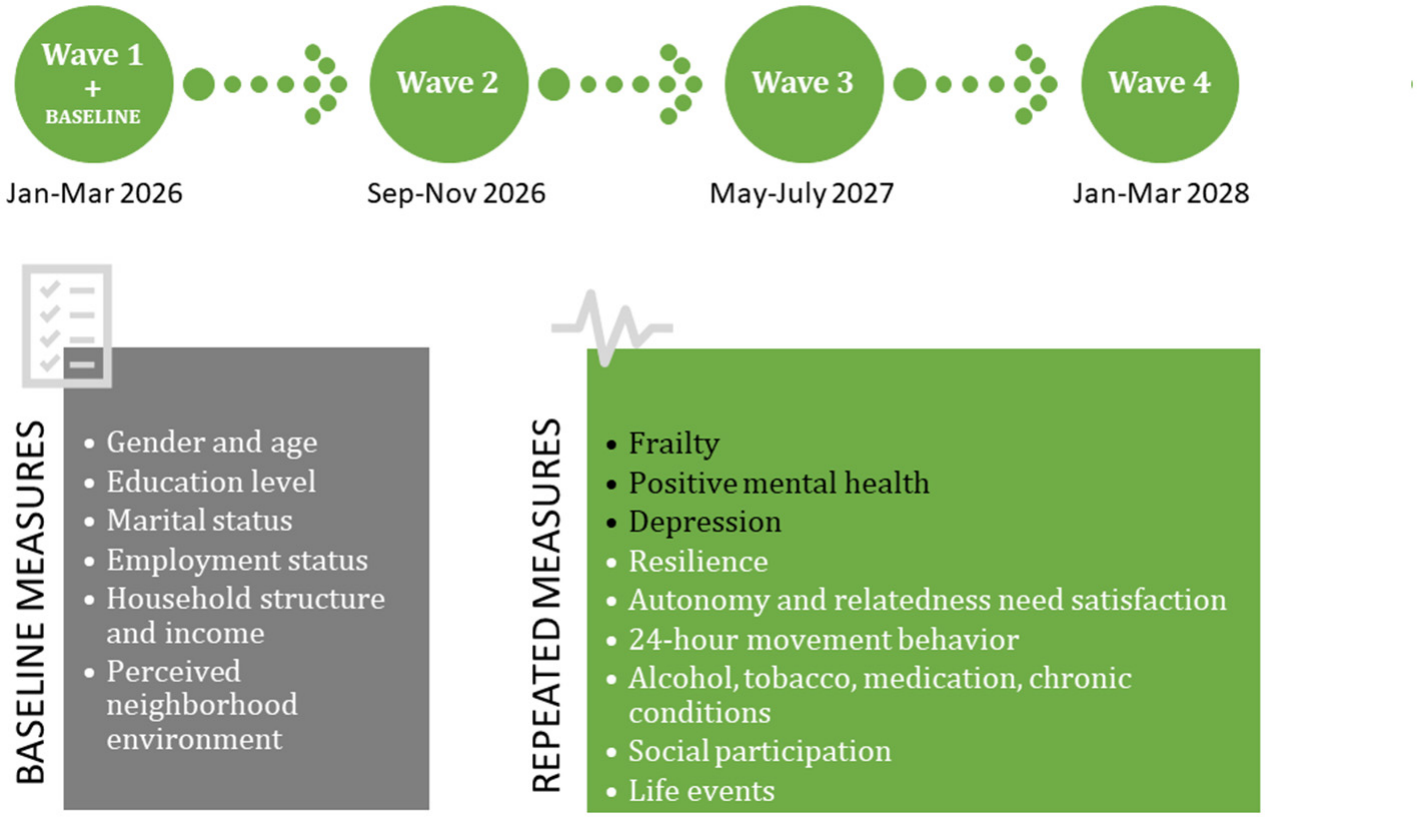
Study design and measurement timeline.

This longitudinal observational study will examine frailty and mental health, together with associated health, social and psychological variables. Repeated measurements will be obtained at four time points over a 27-month period (approximately 8 months apart). The study design and timing of measurements are illustrated in Figure 1.

### 2.2 Study setting

The study will be conducted in the Republic of Slovenia using a nationally sampled population of community-dwelling adults aged 50–84 years. The data, collected at four time points between January 2026 and March 2028, will be stored and analyzed at the Center for Mental Health of the National Institute of Public Health (NIJZ) in Ljubljana.

### 2.3 Participants and sampling

The target population involves community-dwelling residents of the Republic of Slovenia aged 50–84 years. The lower age limit was selected to capture early frailty processes beginning in midlife (5), while the upper limit reduces attrition associated with advanced age while retaining individuals at high risk of frailty progression. Individuals residing in institutional care settings (e.g., nursing homes or other long-term care institutions) will be excluded, as frailty prevalence and associated health characteristics differ substantially between institutionalized and community-dwelling populations (6).

The sample will be prepared by the Statistical Office of the Republic of Slovenia (SURS) using a stratified simple random sampling design. Stratification by statistical region, settlement type, and age group ensures geographic representativeness and enables subgroup comparisons. Approximately equal representation of the three age groups (50–64, 65–74, 75–84 years) is planned to facilitate comparisons across age categories.

### 2.4 Measures

#### Primary measures

Frailty will be assessed using the Tilburg Frailty Indicator (TFI), a 15-item instrument covering physical, psychological and social domains (7). The TFI has demonstrated good reliability and validity, including in community-dwelling older adults in Slovenia (6).

Positive mental health will be measured using the Mental Health Continuum—Short Form (MHC-SF), a 14-item scale assessing emotional, social and psychological wellbeing (8). The instrument has shown good psychometric properties in international studies (8–10).

Depressive symptoms will be assessed using the Patient Health Questionnaire−9 (PHQ-9), a widely used measure of depressive symptom severity (11). The PHQ-9 has demonstrated good reliability and validity, including in Slovenian samples (12).

Additional details on all measures, including item examples and psychometric properties, are provided in Supplementary Material.

#### Psychological, health-related, and social factors

Resilience will be measured using the 10-item Connor–Davidson Resilience Scale (CD-RISC-10), which assesses adaptability, self-efficacy, emotional regulation, optimism, and cognitive focus (13). The scale has demonstrated good psychometric properties in Slovenian adults (14).

Basic psychological need satisfaction of autonomy and relatedness will be assessed using selected subscales from the Basic Psychological Need Satisfaction and Frustration Scale (BPNSFS) (15). The instrument has been shown to be reliable and valid across different cultural contexts (16, 17).

Physical activity, sedentary behavior and sleep (i.e. 24-h movement behavior) will be assessed using the GIB24 questionnaire, a 10-item instrument developed and validated in Slovenia (18).

Additional health-related factors, including alcohol use, tobacco use, medication use, and chronic conditions, will also be assessed using study-specific items. Alcohol use will be measured by self-reported quantity and frequency of drinking in the past 6 months. Tobacco use will be assessed as current smoking status. Medication use will be measured by the number of medications currently taken. Chronic conditions will be assessed using a checklist of six predefined diagnoses, with participants indicating whether they have each diagnosis and whether it was made within the past 6 months or earlier.

Social participation will be assessed through involvement in community and voluntary organizations (e.g., cultural, religious or hobby-based groups), as well as frequency of engagement in leisure-time activities.

Positive (e.g., the birth of a family member, improvements in health, or significant personal achievements) and negative life events (e.g., the death of a loved one, serious illness, or divorce) experienced within the past 6 months will be assessed to capture relevant contextual factors.

#### Baseline measures

Participants will provide information on sociodemographic characteristics, including gender, year of birth, marital status, number of household members, employment status, education level, and household income.

Perceived neighborhood environment (e.g., access to services, green spaces, safety and environmental quality) will be assessed at baseline using study-specific items.

### Timing of measurements

Primary variables, as well as psychological, social, and health-related factors will be assessed at each wave of data collection. These measures refer to recent time periods (e.g., the past month or past 6 months), enabling the analysis of within-person changes over time. Sociodemographic characteristics will be collected at baseline and treated as time-invariant variables in the analyses (see Figure 1).

### 2.5 Recruitment and data collection procedures

In the first wave, all individuals in the sample received a postal invitation package containing a paper questionnaire and an information letter. Participants had the option to complete the questionnaire either in paper form and return it by mail or electronically via an online version accessible through a QR code printed on the questionnaire. The baseline wave of data collection has been successfully completed in March 2026 as planned, and we are currently preparing for the subsequent waves.

Participants were informed at the outset that the study consists of four waves and that they will be invited to participate in subsequent waves. In the first wave, participants were offered the opportunity to voluntarily provide their email address or telephone number if they wish to be contacted by a trained member of the research team via email or telephone for future waves of the study.

Participation is voluntary and may be discontinued at any time without consequences. Completion of the questionnaire will be considered as provision of informed consent.

The estimated time required to complete the questionnaire is approximately 20–25 min, based on pilot testing conducted with a small group of participants. To acknowledge participants' time and effort, those who participate in at least three waves will receive a small symbolic gift in the form of project promotional material. Information about this incentive is clearly stated in the initial invitation.

At the time of publishing this protocol, data collection for the first wave has concluded. A total of 1,650 responses were received, consisting of 1,429 paper questionnaires and 221 electronic submissions, yielding an overall response rate of 33%. The research team is currently compiling and cleaning the database in preparation for subsequent waves.

### 2.6 Statistical analysis

Descriptive statistics will summarize sample characteristics and study variables at each wave. Differences between respondents and non-respondents will be examined to assess potential selection bias. Group differences in categorical variables will be assessed using χ^2^ tests, and differences in continuous variables using *t*-tests or non-parametric alternatives, as appropriate.

Frailty and mental health will be analyzed using validated multi-item instruments. Latent variable modeling approaches will be applied to account for measurement properties. Confirmatory factor analyses will be conducted to evaluate the measurement properties of the constructs across waves, and model fit will be assessed using standard indices such as the Comparative Fit Index (CFI), Tucker–Lewis Index (TLI), Root Mean Square Error of Approximation (RMSEA), and Standardized Root Mean Square Residual (SRMR).

Longitudinal associations between frailty and mental health will be examined using structural equation modeling for panel data across four waves. Random-intercept cross-lagged panel models (RI-CLPM) (19), will be used to examine temporal associations while accounting for both between-person differences and within-person changes over time.

Additionally, health-related, social and psychological variables will be examined as potential explanatory factors in focused longitudinal analyses. Baseline sociodemographic characteristics and other time-invariant variables will be included as covariates in the longitudinal models.

Missing data patterns will be examined and reported for each study wave. The missing data mechanism will be assessed by comparing participants with complete and incomplete data on key demographic and study variables. Primary longitudinal analyses will be conducted using Full Information Maximum Likelihood (FIML), which enables the use of all available data under the assumption that data are missing at random (MAR) (20). To assess the robustness of the findings against potential departures from the MAR assumption, sensitivity analyses using pattern-mixture modeling will be employed. Alternative model specifications, including traditional cross-lagged panel models, will be tested to assess the structural robustness of findings.

### 2.7 Sample size and statistical power

A Monte Carlo simulation was conducted in Mplus 8.8 to evaluate statistical power for the planned longitudinal panel analyses, using RI-CLPM as the primary reference model with four waves of data and 85% retention per wave. The simulation assumed autoregressive effects of 0.50 to 0.60, reflecting moderate temporal stability and consistent with longitudinal cross-lagged analyses reporting similar stability for frailty-related and psychological constructs (21), as well as cross-lagged effects of absolute magnitude 0.07 between frailty and mental health. This value corresponds to a moderate cross-lagged effect according to recent empirical benchmarks for cross-lagged models (22).

With an initial sample size of 1,500 participants and 2,000 Monte Carlo replications, the simulation indicated adequate power to detect the hypothesized cross-lagged effects, with power of approximately 0.83 for the pathway from frailty to subsequent mental health and 0.80 for the pathway from mental health to subsequent frailty. Results suggest that a baseline sample of approximately 1,500 participants, assuming an average retention rate of 85% per wave across four repeated measurements, is sufficient for the planned longitudinal analyses. To promote participant retention across study waves, participants were informed that those who complete at least three waves of data collection will receive a small gift as a token of appreciation. Additionally, participants will be provided with selected insights from preliminary study findings following each wave to keep them informed about the progress of the study and the contribution of their participation. As the last retention measure, participants who consent to future contact will be contacted through their preferred communication channel (email or telephone) for subsequent waves.

To achieve the required baseline sample, 5,000 individuals will be invited to participate in wave 1 from the sampling frame prepared by SURS. Recent national health surveys conducted by the NIJZ have reported response rates of approximately 41% to 67%. However, these surveys were cross-sectional and included broader adult age groups. Because the present study targets older adults and includes four waves of data collection, cumulative attrition is expected. Therefore, a conservative recruitment target was chosen to ensure a sufficient baseline sample for the planned longitudinal analyses.

### 2.8 Attrition and mortality linkage

Additional analyses will compare participants who remain in the study with those who drop out in subsequent waves. To assess potential reasons for attrition in follow-up waves, a one-time secure linkage will be conducted between study data and the official mortality database maintained by the NIJZ. The purpose of this linkage is to determine whether non-participation in follow-up waves is attributable to participant death.

For participants who complete at least one wave and subsequently discontinue participation, information on mortality is analytically relevant because death represents a competing event that may occur prior to the development of frailty or changes in mental health. Death will therefore be treated differently from other forms of missing data due to attrition. The linkage will be conducted in accordance with applicable data protection regulations and secure data handling procedures.

## 3 Discussion

This study is designed to provide a comprehensive longitudinal assessment of frailty and mental health in a nationally sampled population of adults over 50 years. By combining deficit-based indicators, such as depressive symptoms, with positive aspects of mental health, the study adopts a multidimensional perspective that reflects current conceptualizations of frailty as a complex and dynamic process (2).

The longitudinal design, including four repeated measurements over approximately 27 months, enables the examination of temporal associations between frailty and mental health, as well as changes within individuals over time. With multiple waves of data, it becomes possible to examine reciprocal relationships between these constructs and to explore potential mechanisms with appropriate temporal ordering (23). The inclusion of a fourth wave further strengthens the design by allowing assessment of lagged effects across multiple intervals and improving robustness to attrition. This approach allows for a more nuanced understanding of how these constructs co-occur over time, extending previous research that has often relied on cross-sectional or shorter-term designs (24).

From a public health perspective, this study may contribute to a better understanding of the role of positive mental health in relation to frailty in aging populations. Identifying aspects of positive mental health that are linked to changes in frailty may support the development of targeted strategies aimed at promoting healthy aging and reducing the burden of frailty at the population level (21, 24). At the same time, several considerations should be taken into account when interpreting the findings. The follow-up period of approximately 2 years may limit conclusions about longer-term trajectories; however, previous research suggests that transitions in frailty status can occur within similar time frames (5, 25). In addition, as in most longitudinal studies, attrition and selective non-response may affect the representativeness of the sample over time. Planned analyses will therefore examine patterns of attrition and apply appropriate statistical approaches to make use of all available data.

## Data Availability

Findings will be disseminated through peer-reviewed open access publications and presentations at national and international public health conferences.
